# Mechanical Properties of Calvarial Bones in a Mouse Model for Craniosynostosis

**DOI:** 10.1371/journal.pone.0125757

**Published:** 2015-05-12

**Authors:** Mehran Moazen, Emma Peskett, Christian Babbs, Erwin Pauws, Michael J. Fagan

**Affiliations:** 1 Medical and Biological Engineering, School of Engineering, University of Hull, Hull, United Kingdom; 2 UCL Institute of Child Health, London, United Kingdom; 3 Weatherall Institute of Molecular Medicine, University of Oxford, Oxford, United Kingdom; University of Liverpool, UNITED KINGDOM

## Abstract

The mammalian cranial vault largely consists of five flat bones that are joined together along their edges by soft fibrous tissues called sutures. Premature closure of the cranial sutures, craniosynostosis, can lead to serious clinical pathology unless there is surgical intervention. Research into the genetic basis of the disease has led to the development of various animal models that display this condition, *e*.*g*. mutant type *Fgfr2^C342Y/+^* mice which display early fusion of the coronal suture (joining the parietal and frontal bones). However, whether the biomechanical properties of the mutant and wild type bones are affected has not been investigated before. Therefore, nanoindentation was used to compare the elastic modulus of cranial bone and sutures in wild type (WT) and *Fgfr2^C342Y/+^*mutant type (MT) mice during their postnatal development. Further, the variations in properties with indentation position and plane were assessed. No difference was observed in the elastic modulus of parietal bone between the WT and MT mice at postnatal (P) day 10 and 20. However, the modulus of frontal bone in the MT group was lower than the WT group at both P10 (1.39±0.30 vs. 5.32±0.68 GPa; *p*<0.05) and P20 (5.57±0.33 vs. 7.14±0.79 GPa; *p*<0.05). A wide range of values was measured along the coronal sutures for both the WT and MT samples, with no significant difference between the two groups. Findings of this study suggest that the inherent mechanical properties of the frontal bone in the mutant mice were different to the wild type mice from the same genetic background. These differences may reflect variations in the degree of biomechanical adaptation during skull growth, which could have implications for the surgical management of craniosynostosis patients.

## Introduction

The mammalian cranial vault largely consists of five flat bones that are joined together along their edges by soft fibrous tissues called sutures [1,2]. The sutures are designed to give the bones flexibility for birth and to allow the skull to expand and grow as the brain enlarges[3]. Sutures are composites of mesenchymal cells that during development differentiate and deposit extracellular matrix consisting primarily of type I and other collagens as well as various bone-related proteins and proteoglycans [1]. Premature closure of the sutures, or craniosynostosis, is a medical condition that occurs in about 1 in 2,500 births [4,5]. The majority of cases (70%) are non-syndromic *i*.*e*. single suture synostosis, with the remaining instances being syndromic (*e*.*g*. Crouzon and Apert), in which more than one suture fuses and where additional features are present such as midfacial hypoplasia [6]. Syndromic craniosynostosis may result in functional abnormalities of the brain, breathing, feeding and vision unless there is surgical intervention [5,7]. However, even after intervention, some children redevelop raised intracranial pressure requiring further surgical procedures [8,9].

Research to understand the genetic basis and clinical course of craniosynostosis [10] has led to the development of various animal models [11–14]. The Crouzon mouse model, type *Fgfr2* ^*C342Y/+*^ [15,16], is particularly interesting since it has a clear phenotype with features mimicking the clinical features in patients. The coronal sutures (joining the parietal and frontal bones) are most frequently affected, causing a predictable wide and short head shape [17,18]. Coronal sutures in the wild type mouse appear to be close (while never fully fused)at about postnatal day thirty (P30) while in the Crouzon mouse overlapping of the frontal and parietal bone at this suture begins at embryonic stages (E18.5) with full closure at typically about P10 [15]. This model provides an invaluable resource with which to understand the biomechanics of normal and craniosynostotic skulls during postnatal development and to improve surgical reconstruction of this condition in the long term [19–21].

Of particular interest to understand the biomechanics of skull growth, is characterisation of the bone’s key mechanical property, elastic modulus. Various studies have used classical tensile testing or three point bending to quantify the elastic modulus of calvarial bone and sutures in normal skulls [22–30]. Indentation is an alternative method that estimates the elastic modulus based on the area of indentation of an indenter tip, usually manufactured from diamond [31–34]. While the previous methods predict an average modulus value over the sample size tested, the indentation method predicts a value over a much smaller area, typically less than a millimetre in size, and so provides the opportunity to examine the variation in property over the sample [35]. Nanoindentation can be used to examine samples less than 0.1 mm in size, and so is ideal for measuring properties in cranial bone and even sutures of rodents and small animals. To the best of our knowledge no study has compared the mechanical properties of normal and craniosynostotic skulls using indentation methods.

The indentation method was used in this study to test the hypothesis that there is no difference in the mechanical properties (here elastic modulus) of bone and sutures in wild type (WT) and *Fgfr2* ^*C342Y/+*^mutant type (MT) mice during their postnatal development. The specific aims of this study were to quantify: (1) the elastic modulus of frontal and parietal bone in WT and MT mice at ten (P10) and twenty (P20) days postnatal development age; (2) the elastic modulus of the sagittal, coronal and posterior frontal sutures, in WT and MT mice at P10; and (3) the variation of the bone properties with indentation position and plane.

## Materials and Methods

### Sample preparation

WT and MT (*Fgfr2* ^*C342Y/+*^) mice at P10 and P20 were used for this study. In total twenty-one mice were examined in this study—see Table 1 for a detailed breakdown of the samples and the study design. While a higher number of specimens would have increased the power of the study, it was decided that for the purpose of this investigation twenty-one specimens (*i*.*e*. five specimens in each group, plus one for indentation in a different plane) was sufficient to address the hypothesis of this study within the framework of Replacement, Reduction and Refinement (3Rs) for humane animal research.

**Table 1:** Summary of the different experiments with the total number of each animal type used and the number tested in each experiment.

		P10		P20	
Experiment		WTn = 5	MTn = 5	WTn = 6	MTn = 5
1: frontal and parietal bone properties		5	5	5	5
2: sagittal, coronal and posterior frontal suture		5	5	-	-
3: variation in bone properties	a) with indentation position	-	-	1	-
	b) with indentation plane	-	-	1	-
	c) in suture properties with age	-	-	1	-

**Fig 1:**
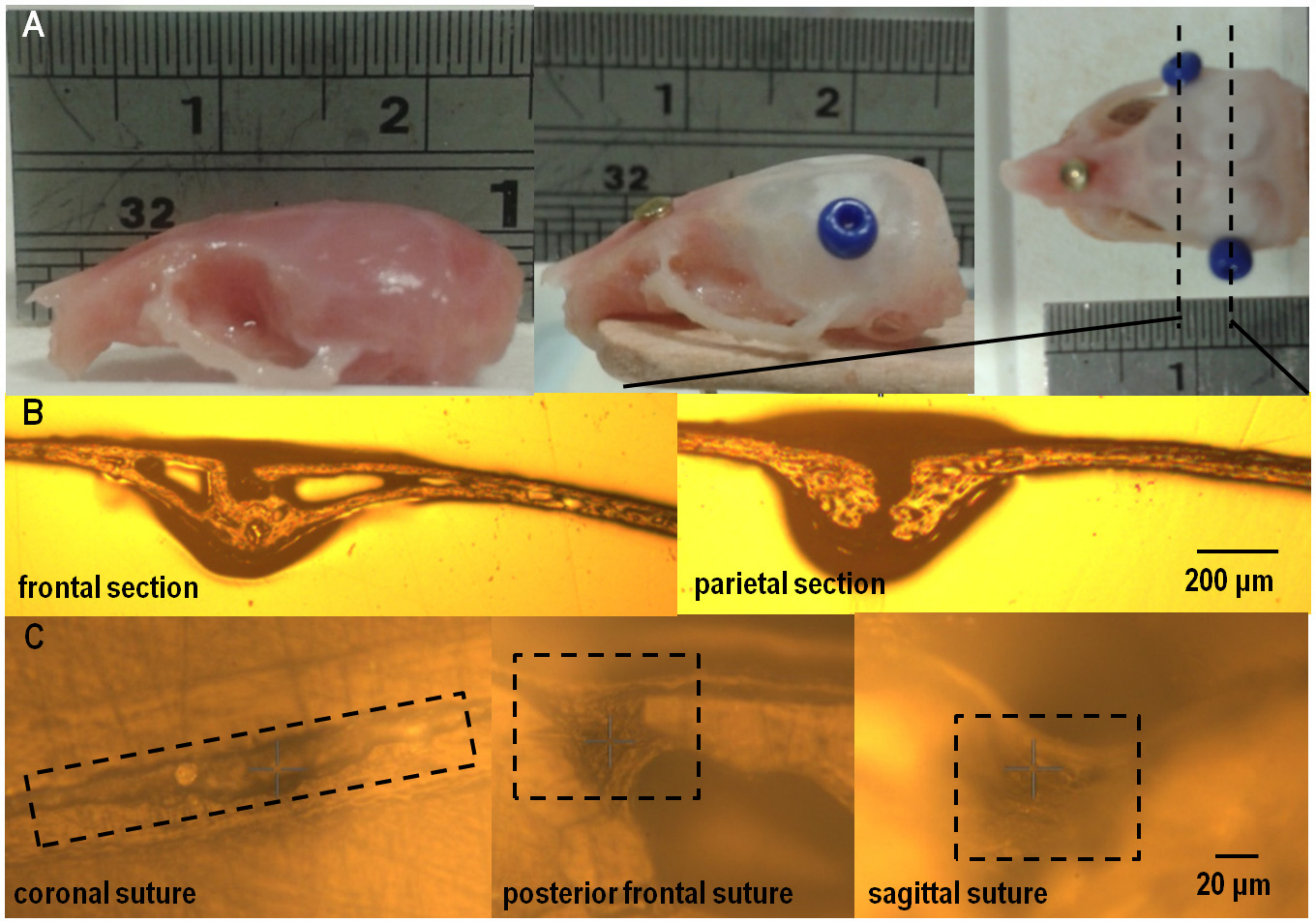
(A) sample preparation, (B) coronal sections through frontal and parietal bones of and (C) coronal, posterior frontal and sagittal suture from left to right in three different WT P20 mice.

All animals were bred from the same genetic background, and polymerase chain reaction (PCR) was performed to identify MT mice, with samples kept frozen until preparation for indentation. Approximately 15h prior to preparing the samples for nanoindentation, the skulls were defrosted and kept in phosphate buffered saline (PBS, pH 7.2, Sigma-Aldrich, MO, USA). The brain, periosteum and dura mater were removed to ensure good fixation at the bone-resin interface. Each cranium was fixed horizontally to a rectangular piece of wood (10×110×1 mm) using a small needle inserted through the nasal bone into the wood. Two small beads (ca.1 mm in length and diameter) were glued using tissue adhesive (B. Braun Medical Ltd., Germany) to the skull. These were placed away from the region of the interest, onto (1) the parietal bone—where the middle of the sagittal suture was selected in the sagittal plane; and (2) the frontal bone—where the middle of the coronal suture was selected in the sagittal plane. These were used as markers to facilitate cutting of the samples in the correct planes (Fig 1A). Samples were then mounted in cold cure epoxy resin (Buehler, Germany). After 24h, the samples were cut and polished in the coronal plane (Fig 1B), first at the parietal section and then at the frontal section. One WT sample at P20 was also cut and polished in the sagittal plane, at less than 1 mm adjacent to the sagittal suture.

Samples were first polished using silicon carbide paper (320,600, 1200 and 2500 grit), and the sutures were indented (Fig 1C). Then, final polishing using aluminium oxide slurries (0.3 and 0.05μm particle size) on neoprene cloth (Buehler, Germany) was performed, and the bones were indented. The sutures were indented before second stage polishing as our initial testing showed that polishing with slurries could damage (wash away) the sutures. The same preparation procedure was followed for each test. The polishing protocol on the bone led to a final surface roughness of ca. 0.2 μm. During the testing it was ensured that the indentation depth was always an order of magnitude larger than this roughness [36].

### Indentation

All indentations were performed at room temperature using a Berkovich diamond tip. The tip was mounted on a CSM indenter (CSM Instruments, Switzerland) that was set up on a vibration-free station (Kinetic Systems Inc., MA, USA). A Berkovich tip was selected because it has been widely used previously for the indentation of bone (*e*.*g*. [36,37]), although less often for the indentation of tissues such as cartilage [37]. While a larger diameter (*e*.*g*. >20 μm) spherical tip allows a larger contact area to test tissue level properties [38], due to the extremely small width and depth of some of the sutures (see Fig 1C) it was decided to use the Berkovich tip for all the measurements.

Samples were kept moist during indentation (by soaking for 10 min after each 10 min of testing in PBS,pH 7.2). Parietal and frontal bones were indented within a 300μm zone adjacent to the sagittal and posterior frontal sutures, while the sutures themselves were indented along their lengths. In those cases where the suture was not entirely patent along its length, only the soft tissue in the patent regions was tested. A minimum spacing of 10 μm between the indentations was ensured [36]. The mean and standard deviation of five indentations was reported in each case. Bone was indented under displacement-control to a depth of 2.5 μm at 120 mN/min followed by 20 s hold [39]. The sutures were indented under load-control to a load of 0.1 mN at 1 N/min followed by 2 s hold [40]. The aforementioned holding times were used to reduce the effect of tissue viscoelasticity. At the same time longer holding times for the sutures were not practical due to large displacements that could lead to contact with the underlying bone. The unloading rates were the same as the loading rates in both cases.

The elastic modulus was calculated using the standard Oliver-Pharr method [31]. In brief, first reduced elastic modulus is calculated from:

$$E_{r}\;=\;\frac{\sqrt{\pi }S}{2\sqrt{A_{c}}}$$

where S is the unloading stiffness calculated as the initial slope (slope at 95%) of a polynomial function fitted over 95–20% of the unloading curve, and A_c_ is the contact area.

The tissue elastic modulus, E_s_ is then estimated from:

$$\frac{1}{E_{r}}\;=\;\frac{1-\upsilon_{s}^{2}}{E_{s}}+\;\frac{1-\upsilon_{t}^{2}}{E_{t}}$$

where ʋ_s_ and ʋ_t_ are the Poisson’s ratio of the tissue and indenter tip, and here assumed to be 0.3 (for both bone and suture [26,27]) and 0.07 respectively; E_s_ and E_t_ are the elastic moduli of the tissue and the indenter tip, here 1140 GPa (from the manufacturer’s data).

The indentation results are sensitive to various factors such as the indenter control parameters [41], and sample preparation [36]. Therefore, several sensitivity tests were conducted before choosing the parameters that were used in this study (see S1 and S2 Tables). Note also that the Poisson's ratio of the suture material is not known, but is likely to be higher than 0.3 in which case, the data presented need to be multiplied by a factor of 0.92 (for a value of 0.4) or 0.82 (for a value approaching 0.5) based on the previous equations.

### Tests

Three main tests using the same samples were performed to address the aims of the study (see Table 1):

1. the frontal and parietal bones of P10 and P20 in WT and MT mice were indented at the coronal plane (5 tests for each case);
2. the sagittal, coronal and posterior frontal sutures were indented in the P10 WT and MT samples in the coronal planes (5 tests for each case);
3. three additional tests were performed in the WT mice: (a) a P20 sample was indented at several positions across the parietal bone in the coronal plane to understand the variation due to the indentation position; (b) a P20 sample was indented in several positions across the sagittal plane, to understand the variation due to the indentation plane; and (c) a P20 sample was indented across the sutures.

### Statistical analysis

Statistical analysis was performed in SPSS (IBM SPSS v19, NY, USA). A dependent (paired) t-test was used to compare the bone and suture data within the WT and MT groups. Independent (two sample) t-tests were used to compare bone and suture data between WT and MT. The significance level was set at *p*<0.05. Considering that sample size in this study was small for each group, results of normality and homogeneity tests were considered with caution and both parametric and equivalent non-parametric tests were performed; but reassuringly both tests led to the same results regarding significance for all comparisons.

### Ethics statement

All the protocols regarding use of laboratory animals were approved by the University College London Committee on Animal Research.

## Results

### Frontal and parietal bones

**Fig 2:**
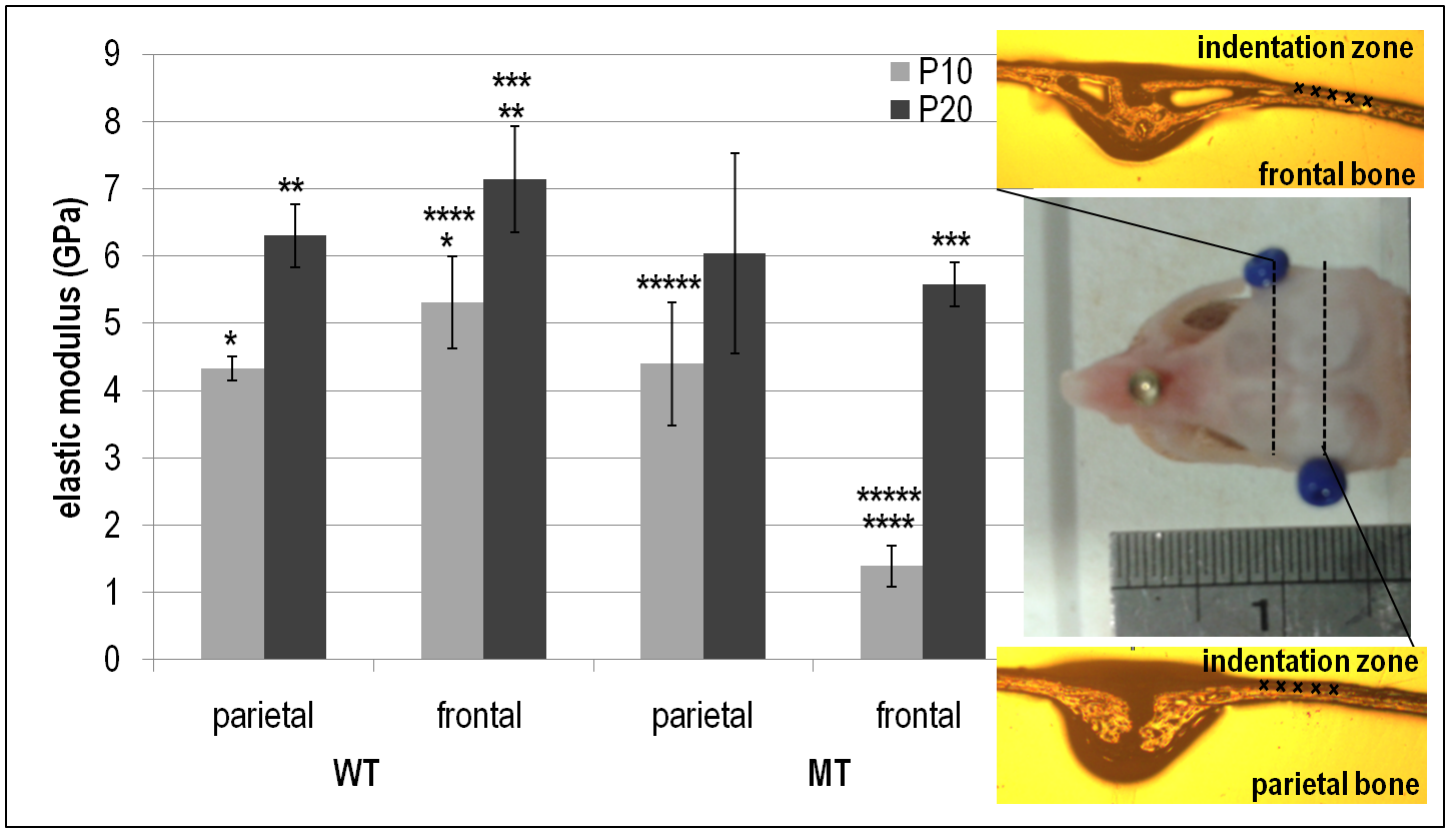
Comparison between elastic modulus of the frontal and parietal bones at P10 and P20 for WT and MT mice. * highlight statistical significance between corresponding groups (*p*<0.05).

Considering the properties of the frontal and parietal bones within the WT and MT groups (Fig 2), the P20 modulus values were higher than the P10 values in all cases. In the WT group the elastic modulus of the frontal bone was higher than parietal bone for both P10 (5.32±0.68 vs. 4.33±0.18 GPa; *p*<0.05) and P20 (7.14±0.79 vs. 6.30±0.47 GPa; *p*<0.05—Fig 2). By contrast, in the MT group the elastic modulus of frontal bone was lower than parietal bone at both P10 (1.39±0.30 vs. 4.40±0.92 GPa; *p*<0.05) and P20 (5.57±0.33 vs. 6.04±1.49 GPa; n.s.).

**Fig 3:**
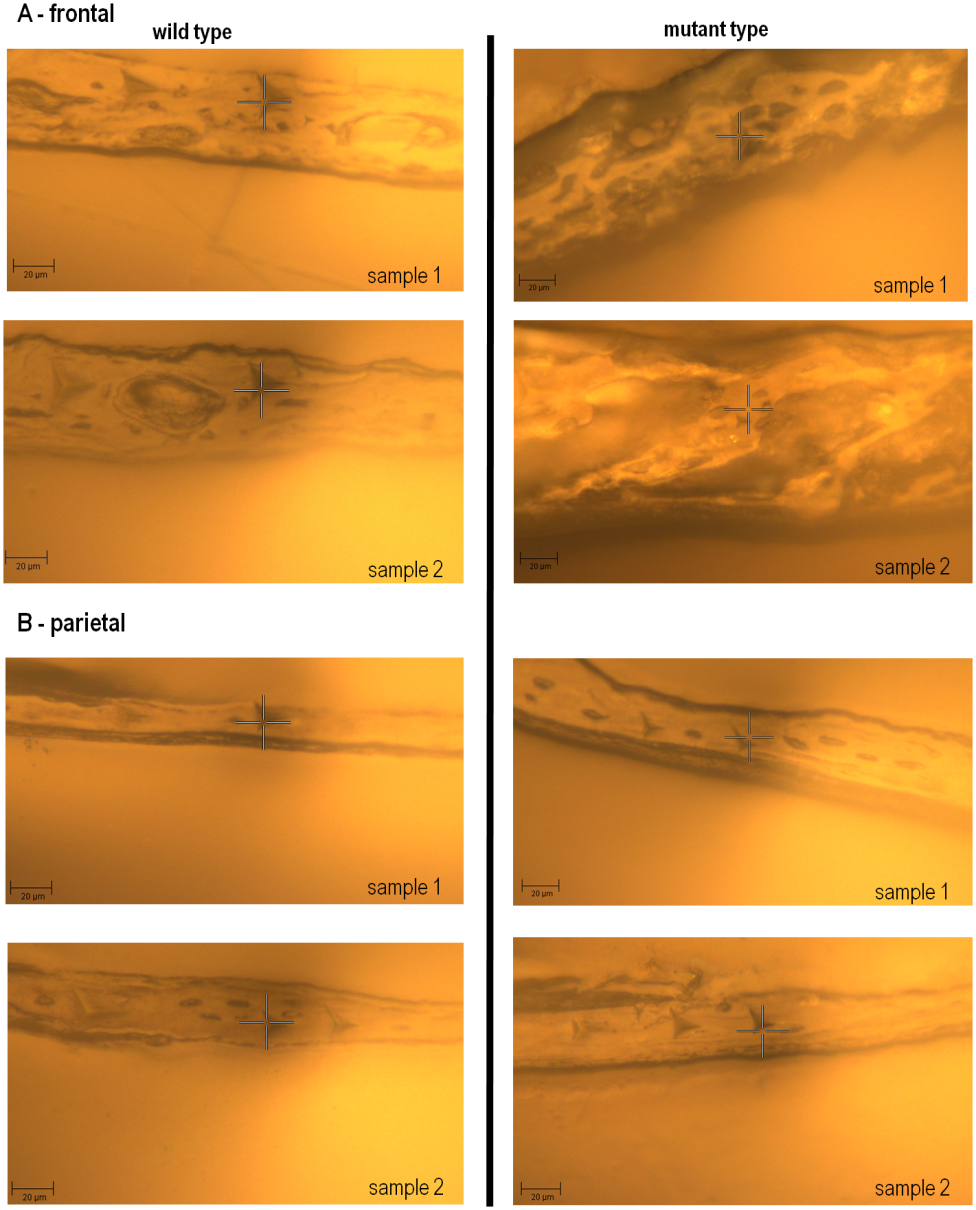
Visual comparison between the frontal (A) and parietal (B) bone in the WT and MT mice at P10 in two samples from each group.

Comparing the properties of the frontal and parietal bones between the WT and MT groups shows that the elastic modulus of frontal bone in the MT group was lower than the WT group at both P10 (1.39±0.30 vs. 5.32±0.68 GPa; *p*<0.05) and P20 (5.57±0.33 vs. 7.14±0.79 GPa; *p*<0.05). However, there was no statistically significant difference in the elastic modulus of parietal bone between the WT and MT at P10 (4.33±0.18 vs.4.40±0.92 GPa) or P20 (6.30±0.47 vs. 6.04±1.49 GPa)—see Fig 2. In fact, the frontal bone was *visually* much more porous, showing less trabecular bone and larger lacunae, at P10 in the MT group comparing to the WT group while the parietal bone was visually similar between the MT and WT (Fig 3). Note that it was not possible to quantify the differences in porosity.

### Sagittal, coronal and posterior frontal suture

**Fig 4:**
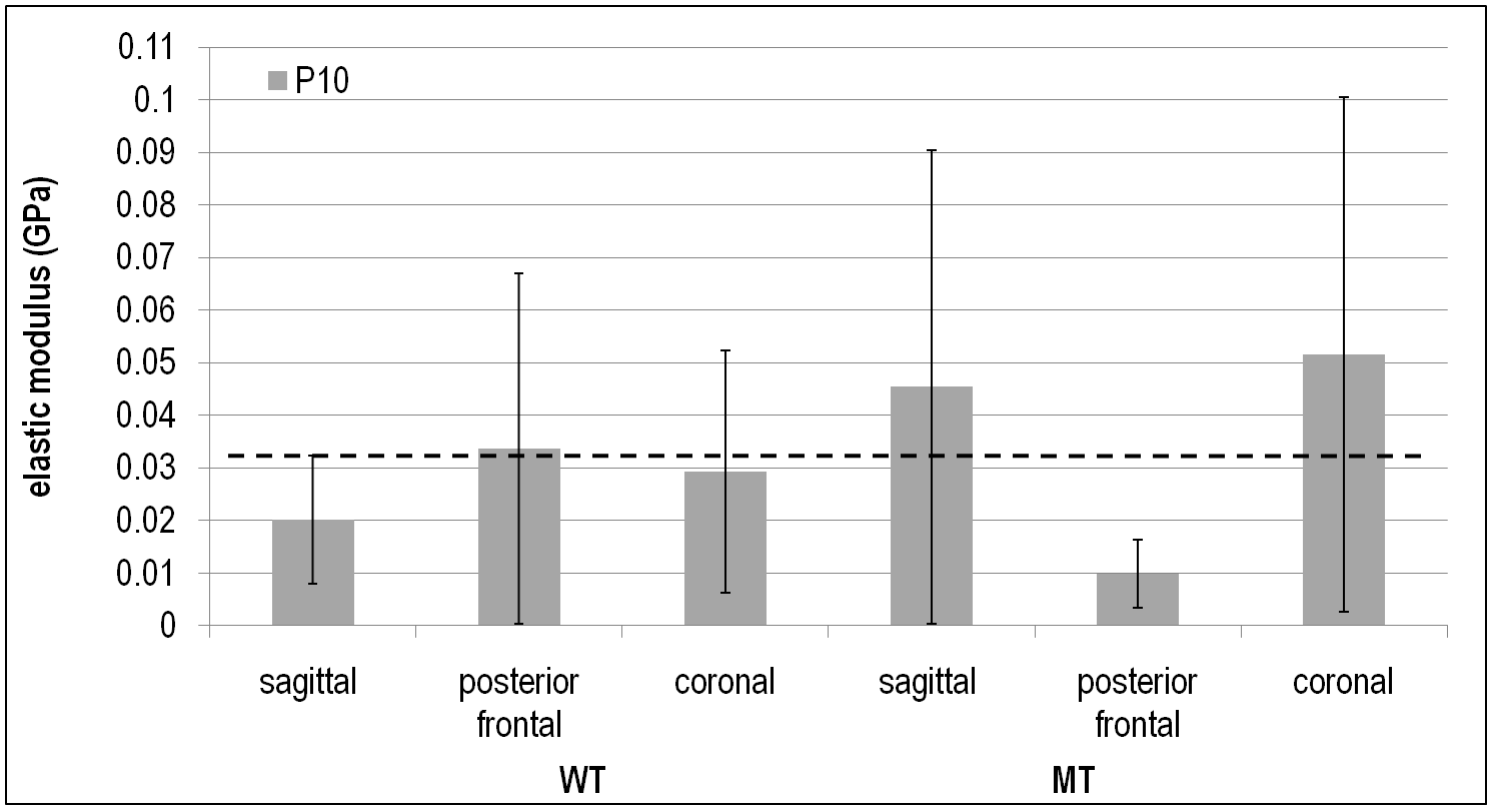
Comparison between the elastic moduli of the sagittal, posterior frontal and coronal sutures at P10 between WT and MT group. The dashed line shows the average of all data.

The standard deviations of the elastic modulus that were observed in the sutures were considerably higher than those observed in the bone (Fig 4). No statistically significant difference was found in the properties of the sagittal, coronal and posterior frontal sutures with and within the WT and MT groups there was. Thus, the elastic moduli of all sutures in both the WT and MT samples ranged from 0.001–0.116 GPa with an average of 0.032±0.032 GPa.

### Variation of the properties due to the indentation position, plane and age

**Fig 5:**
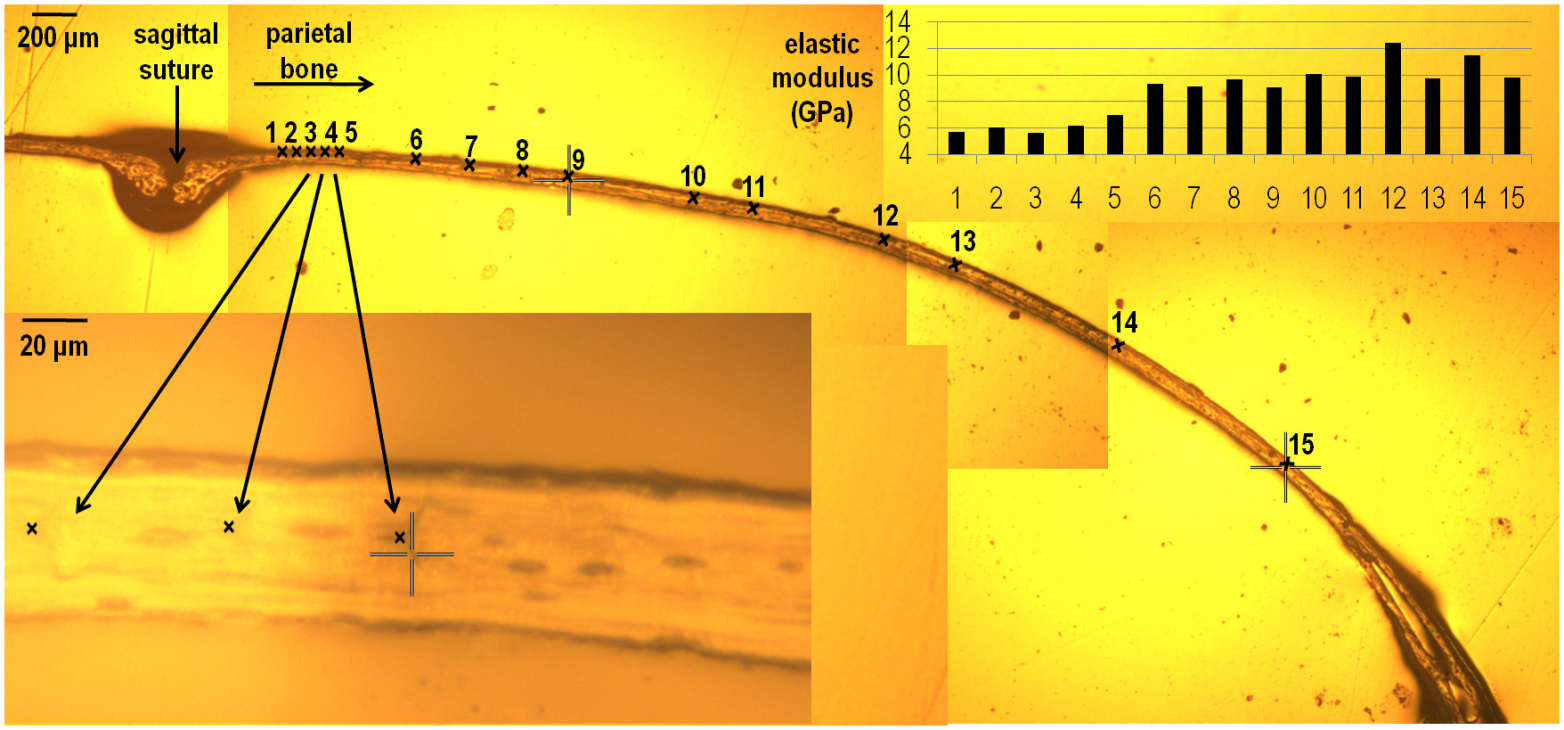
Variation in the elastic modulus of the parietal bone in the coronal plane in a P20 WT mouse.

**Fig 6:**
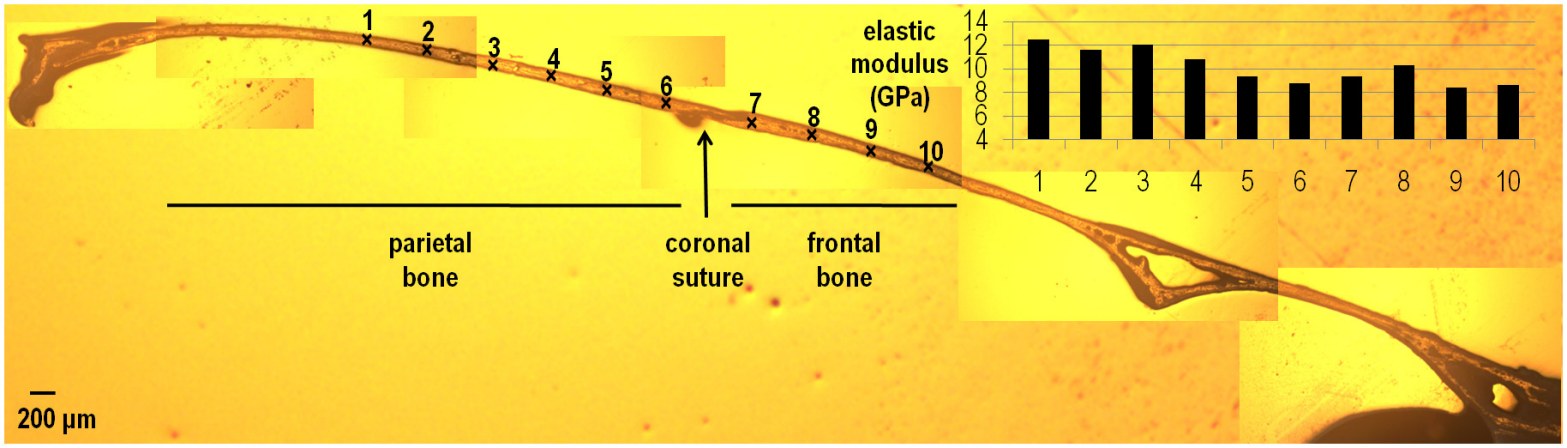
Variation in the elastic modulus of the frontal and parietal bone in the sagittal plane in a P20 WT mouse.

As the indentation position moved laterally along the bone in the coronal plane from the sagittal suture (see points no 1–15 in Fig 5), the elastic modulus of parietal bone gradually increased. In the sagittal plane, the elastic modulus of parietal bone gradually decreased from 12.5 GPa to less than 9 GPa as the indentation position approached the coronal suture (see points no 1–6 in Fig 6). By comparison, the elastic modulus of frontal bone, moving from the coronal suture anteriorly, gradually increased and then decreased again (see points 7–10 in Fig 5). Comparing the elastic modulus of frontal versus parietal bone based on the closest indentations to the coronal suture, shows that frontal bone maybe marginally stiffer (9.4 vs. 8.7 GPa see Fig 6). The elastic modulus of the sutures in the P20 sample were 0.006, 0.01 and 0.003 GPa for the sagittal, coronal and posterior frontal suture respectively. These data were within the range of data recorded from the P10 samples.

## Discussion

Understanding the genetic basis of craniosynostosis has led to the development of various animal models that have enhanced our understanding of the pathogenesis of forms of this condition. However, the impact of these genetic mutations on the inherent properties of the bone and sutures has not been investigated before.

### Frontal and parietal bones

Within the WT group, frontal bone consistently showed a higher elastic modulus compared to the parietal bone. A similar finding was reported recently by Wang et al. [30] on 1.5 year old infants. However, the difference in the properties of the two bones decreased during the development from P10 to P20 from 23% to 13%. This is interesting as the frontal bone arises from the neural crest, while the parietal bone originates from the mesoderm[42], which may be related to the difference. It appears however that the properties of parietal and frontal bone converges to the same value during postnatal development regardless of their origins. This would imply a slower rate of ossification and/or mineralisation for the frontal bone(see also [43]).

Comparing the properties of the frontal and parietal bones between the WT and MT groups, while there was almost no difference between the properties of the parietal, the frontal was significantly less stiff in the MT comparing to WT. At P10, the MT value was just 26% of the WT value, at P20 it was 78%. The fact that frontal bone was *visually* much more porous at P10 in the MT group comparing to the WT group (Fig 3) explains that the differences in the elastic modulus of the frontal bone in the WT and MT at P10 is probably a reflection of not only differences in the tissue properties, but also variations in structural properties (bone architecture). At the same time since no structural (visual) difference between the frontal of WT and MT was observed at P20 yet the frontal had a lower elastic modulus, it is likely that finding of lower elastic modulus at P10 for frontal in MT versus WT is not solely due to the structural difference of the bone atP10 but also due to the underlying biological differences. The finding of a less stiff frontal bone in the MT individuals is interesting from several aspects. From a biomechanical point of view, it may reflect continuing adaptation to an adjacent fast closing (fused) coronal suture in the presence of a rapidly growing brain. From a biological point of view, it raises the question as to why the parietal bone is not responding in a similar way. The answer may lie in the cellular biology and embryological differences in the frontal and parietal bone [42,44,45], or it is probable that a less stiff frontal in MT is a combined effect of biological and biomechanical factors.

### Sagittal, coronal and posterior frontal suture

The elastic moduli of the sutures measured in this study had high standard deviations. This could be due to various reasons *e*.*g*. tissue handling and impact of humidity [35], testing the sutures across their cross section as opposed to a more specific indentation site [40], or there is a naturally wide variation in properties. Therefore, the suture data presented in this study should be considered with caution and as preliminary data for further future investigations. Noting that caveat, the current data suggest: (1) the biomechanical properties of sutures are similar across the different regions of the skull (see also [30]); and (2) the coronal suture in MT individuals is similar to that of the WT, while the speed of bone growth (or sutural fusion) is different in MT (*Fgfr2* ^*C342Y/+*^) compared to the WT group.

### Variation of the properties due to the indentation position, plane and age

Varying the indentation position in the parietal bone across a coronal section showed a gradual increase in the elastic modulus of the bone from the medial (sagittal suture- about 6 GPa) to the lateral aspect of the skull (about 10 GPa—Fig 5). This was analogous and consistent with an increase in the elastic modulus of parietal bone measured adjacent to the sagittal suture during development *i*.*e*. at P10 and 20. In fact a similar pattern, *i*.*e*. a gradual increase in the elastic modulus of the bone, was observed as the indentation position moved away from the coronal suture in the sagittal plane (Fig 6). In brief, it appears that the elastic modulus of the cranial bones gradually increases during postnatal development and converges to typically 9–12 GPa in mature bone or adults. This agrees with values reported previously in the literature [23,26,46].

Given the wide range of elastic modulus values that was recorded in the sutures in both WT and MT P10 mice, it was interesting that the elastic modulus of the sutures in the single P20 sample were within the range of data recorded for the P10 mice. This finding itself was similar to previous studies that tested elastic modulus of sutures during development [24,27]. Possibly this reflects the fact that the suture properties are constant (in the range of 0.03 GPa) regardless of age. In fact, this value is close to the results of Henderson et al. [27] who reported an elastic modulus of 0.004 to 0.08 GPa for the sagittal suture of rats age P2–60. The average value of all their data (see Fig 6a of [27]) was about 0.022 GPa, comparable to 0.032 GPa that was found in this study. This was also interesting since the current study and that of Henderson et al.[27] both used different methodologies, *i*.*e*. nanoindentation versus tensile testing, yet found similar results.

### Clinical relevance

From a clinical point of view, craniofacial and neurosurgeons perhaps need to be aware of the variation in bone properties in different regions of the calvaria when performing calvarial reshaping in craniosynostosis patients. For the same thickness of material, the lower elastic modulus of frontal bone compared to parietal bone would result in greater flexibility and strain. Therefore, mixing the two types during reshaping may free or over-constrain the natural growth of the underlying brain and affect subsequent remodelling and integration of the different sections. Further studies are required to investigate the potential impact of bone properties on the outcome of surgery using predictive computational tools such as finite element analysis [20,21].

### Limitations

Perhaps the key limitation of this study is that the number of specimens in each group is only five. This perhaps has had minimal effect on the major finding of this study regarding the bone data where a distinct difference is observed between the frontal bone elastic modulus in the WT and MT group. Of some concern is the high variability observed in the suture data, but other studies have found similar variability. For example, Henderson et al. [27] tested the sagittal suture of seventy rats at various ages and also found a large variability for the elastic modulus of the suture, in the range of 0.004 to 0.08 GPa, with wide ranges at each age group. Therefore, it is possible that sutures naturally have a wide variation in their properties, or their high viscoelasticity combined with very small but variable sizes, makes measurement of their properties especially challenging. In any case further studies are required to investigate suture properties. It must also be noted that present study comment on the higher porosity of the frontal bone in the MT group at P10 is just a qualitative observation and further three dimensional studies based on computed tomography are required to quantify the bone porosity.

## Conclusions

Findings of this study suggest that the mechanical properties of frontal bone are, at least at the early stages of postnatal development, different between the wild type (WT) and *Fgfr2* ^*C342Y/+*^mutant (MT) mice. By contrast, the mechanical properties of parietal bone and sutures are more similar between the WT and MT mice tested in this study. From a clinical point of view, craniofacial and neurosurgeons perhaps need to be aware of this when performing calvarial reshaping in craniosynostosis patients, since the mechanical properties of the various bones could be significantly different in different regions of skull. This could have an impact on the outcome of the surgical procedure and require further investigations.

## Supporting Information

S1 Table Sensitivity of the Elastic modules of the bone to the indentation force (depth) and speed. Data obtained from the parietal bone of a mutant specimen at postnatal day 20. (DOC)

S2 Table Sensitivity of the Elastic modules of the suture to the indentation force (depth) and speed. Data obtained from the sagittal suture of a mutant specimen at postnatal day 20. (DOC)
