# Supplementary material for: Mechanical Properties of Calvarial Bones in a Mouse Model for Craniosynostosis
Source: PLoS One. 2015 May 12;10(5):e0125757. doi: 10.1371/journal.pone.0125757 (PMC4429024; doi:10.1371/journal.pone.0125757)
Supplement: S1 Table — (DOC) [file pone.0125757.s001.doc]

**Table S1: Sensitivity of the Elastic modules of the bone to the indentation force (depth) and speed. Data obtained from the parietal bone of a mutant specimen at postnatal day 20.**

| Test 1 |  |  |
| --- | --- | --- |
| Number of repeats | Force - 10 mN,  Speed - 300 mN/min |  |
|  | Depth (μm) | E (GPa) |
| 1 | 1.056 | 6.87 |
| 2 | 0.867 | 9.13 |
| 3 | 1.223 | 5.97 |
| 4 | 1.176 | 5.34 |
| 5 | 1.299 | 5.96 |
|  | Average | 6.65 |
|  | Standard deviation | 1.49 |
| Test 2 |  |  |
|  | Force - 20 mN  Speed - 600 mN/min |  |
|  | Depth (nm) | E (GPa) |
| 1 | 2.187 | 4.41 |
| 2 | 2.243 | 4.36 |
| 3 | 2.381 | 4.28 |
| 4 | 2.516 | 4.18 |
|  | Average | 4.30 |
|  | Standard deviation | 0.09 |
| Test 3 |  |  |
|  | Force - 40 mN  Speed - 1200 mN/min |  |
|  | Depth (nm) | E (GPa) |
| 1 | 3.251 | 4.29 |

Bone data presented here suggest that elastic modulus of bone was sensitive to the indentation load and depth. Therefore, indentation load was increased in three steps to understand at what loading rate and indentation depth, elastic modulus was converged. Data highlighted that at indentation depth of above 2 μm, elastic module was converged (Test 1-3). Therefore, indentation on the bone was performed under displacement-control to a depth of 2.5 μm at 120 mN/min. The chosen speed was based on the work of Wolfarm et al. [39].
