# Supplementary material for: Mechanical Properties of Calvarial Bones in a Mouse Model for Craniosynostosis
Source: PLoS One. 2015 May 12;10(5):e0125757. doi: 10.1371/journal.pone.0125757 (PMC4429024; doi:10.1371/journal.pone.0125757)
Supplement: S2 Table — (DOC) [file pone.0125757.s002.doc]

**Table S2: Sensitivity of the Elastic modules of the suture** to the indentation force (depth) and speed. Data obtained from the sagittal suture of a mutant specimen at postnatal day 20.

|  | Force (mN) | Speed (mN/min) | Depth (μm) | E  (GPa) |
| --- | --- | --- | --- | --- |
| Test 1 |  |  |  |  |
| 1 | 10 | 300 | 4.201 | 1.630 |
| 2 | 10 | 300 | 2.400 | 0.840 |
| 3 | 10 | 300 | 2.000 | 0.589 |
| 4 | 10 | 300 | 1.855 | 0.505 |
| Test 2 |  |  |  |  |
| 1 | 5 | 30 | 1.915 | 1.629 |
| Test 3 |  |  |  |  |
| 1 | 0.6 | 18 | 2.603 | 0.933 |
| 2 | 0.6 | 18 | 1.482 | 1.339 |
| 3 | 0.6 | 18 | 2.854 | 0.559 |
| Test 4 |  |  |  |  |
| 1 | 0.3 | 3 | 3.319 | 0.013 |
| 2 | 0.3 | 3 | 4.249 | 0.008 |
| 3 | 0.3 | 3 | 3.932 | 0.009 |
| 4 | 0.3 | 3 | 3.188 | 0.012 |
| Test 5 |  |  |  |  |
| 1 | 0.1 | 1 | 3.475 | 0.002 |
| 2 | 0.1 | 1 | 3.331 | 0.006 |
| 3 | 0.1 | 1 | 5.708 | 0.002 |
| 4 | 0.1 | 1 | 3.505 | 0.009 |
| 5 | 0.1 | 1 | 2.959 | 0.009 |

Suture data presented here suggest that elastic modulus of sutures were sensitive to the indentation load. High indentation load and speed resulted in penetration of the indentation tip through the sutures and reaching the underlying bone as the indentation load and speed decreased from 10 to 0.1 mN and 300 to 1 mN/min (Test 1-5) elastic modulus converged at 0.3-0.1 mN to about 0.01 GPa. Therefore, indentation on the suture was performed under load-control to a load of 0.1 mN at 1 mN/min. The chosen speed was based on the work of Leong and Morgan [40].
